# Examining young people’s views and understanding of traffic light and physical activity calorie equivalent (PACE) food labels

**DOI:** 10.1186/s12889-023-16019-6

**Published:** 2023-06-14

**Authors:** Natalia Iris, Fehmidah Munir, Amanda J. Daley

**Affiliations:** 1 Centre for Lifestyle Medicine and Behaviour, School of Sport, Exercise and Health Sciences, Loughborough University, Loughborough, LE11 3TU UK; 2 School of Sport, Exercise and Health Sciences, Loughborough University, Loughborough, LE11 3TU UK

**Keywords:** Food labelling, PACE labelling, Calories, Food choice, Adolescents, Children

## Abstract

**Background:**

Childhood obesity is a public health challenge in many countries. Food labelling may help children make healthier food choices. Food is typically labelled using the traffic light label system but this is complex to understand. Physical activity calorie equivalent (PACE) labelling may be easier for children to understand and more appealing because it contextualises the energy content of food/drinks.

**Methods:**

A cross-sectional online questionnaire was completed by 808 adolescents aged 12–18 years in England. The questionnaire investigated participants’ views and understanding of traffic light and PACE labels. Participants were also asked about their understanding of the meaning of calories. The questionnaire explored participants’ views about the potential frequency of use of PACE labels and their perceived usefulness in influencing purchasing and consumption decisions. Questions that explored participants’ views about the possible implementation of PACE labelling, preferences for food settings and types of food/drinks they may like such labelling implemented, and whether PACE labels would encourage physical activity were included. Descriptive statistics were explored. Analyses assessed associations between variables and tested differences in the proportions of views about the labels.

**Results:**

More participants reported PACE labels as easier to understand than traffic light labels (69% vs 31%). Of participants who had seen traffic light labels, 19% looked at them often/always. Forty-two percent of participants would look at PACE labels often/always. The most common reason why participants never/would never look at food labels is because they are not interested in making healthy choices. Fifty-two percent of participants said PACE labels would make it easier for them to choose healthy food and drinks. Fifty percent of participants reported PACE labels would encourage them to be physically active. It was perceived that PACE labels could be useful in a range of food settings and on a range of food/drinks.

**Conclusions:**

PACE labelling may be easier for young people to understand and more appealing/useful to them than traffic light labelling. PACE labelling may help young people choose healthier food/drinks and reduce excess energy consumption. Research is now needed to understand the impact of PACE labelling on food choice among adolescents in real eating settings.

**Supplementary Information:**

The online version contains supplementary material available at 10.1186/s12889-023-16019-6.

## Background

Childhood obesity is a major public health challenge [1]. In England, childhood obesity prevalence has increased, with around 40% of children now living with overweight or obesity by the time they leave primary school [2]. Around a third of children are doing less than an average of 30 minutes physical activity per day [3]. Having excess weight increases the risk of long-term conditions such as cardiovascular disease, cancer and diabetes [1]. Childhood obesity has also been linked with negative psychological effects such as low self-esteem and depression [4].

**Fig. 1:**
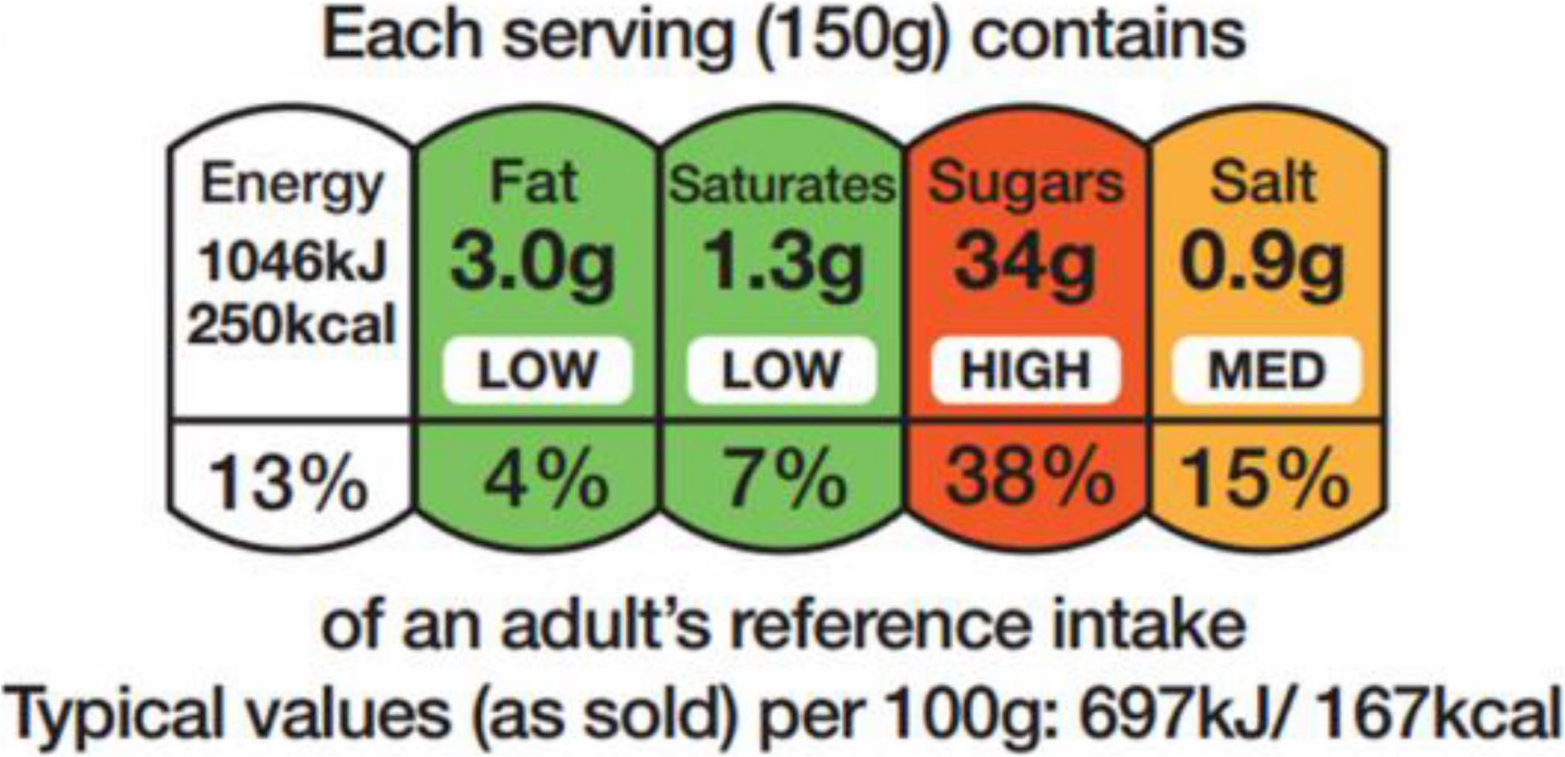
Example of a TLL Source: Food Standards Agency (https://www.food.gov.uk/safety-hygiene/check-the-label)

Evidence suggests that the average adolescent is consuming an excess of energy, typically, from sugary drinks, confectionary and cakes [5]. Eating out of the home has been linked with unhealthy food choices [6]. One strategy to help promote healthier eating and drinking is nutrition labelling. A common food label on packaged food and drinks in the UK (United Kingdom) and other countries is the traffic light label (TLL) (see Fig. 1) [7, 8]. In the UK, the TLL uses colour coding to display whether a food or drink is high, medium or low in fat, saturated fat, sugar and salt [7]. The TLL also shows energy information [7].

Recognising that eating out of the home can drive the overconsumption of calories, governments in several countries, including the UK, have made it mandatory for large businesses in the out of home sector (such as restaurants, cafés and takeaways) to have calorie labelling [9, 10].

**Fig. 2:**
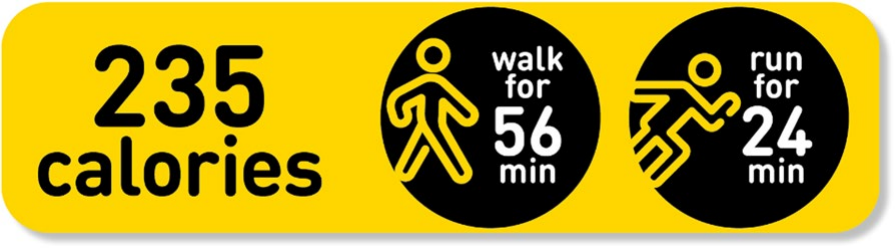
Example of a PACE label. Source: Loughborough University/the Authors

Though nutrition labelling is considered a way to promote healthy eating, it is unclear whether it influences food purchasing or consumption [11, 12]. Nutrition labelling may not be effective, or not effective as it could be, because it displays information that is hard to understand and interpret (e.g. number of calories) [13]. This may be particularly true for children as current food labelling approaches require complex thought processes. Until early adolescence, children are unable to think hypothetically [14]. Therefore, children may struggle to understand the context of eating/drinking unhealthily such as how it relates to energy balance. Given this concern, an alternative approach to food labelling that may be more appealing to young people is physical activity calorie equivalent (PACE) food labelling (see Fig. 2). This labelling aims to contextualise the energy content of food/drinks by showing the number of minutes or miles/kilometres of physical activity equivalent to the calories contained in the item. As well as being a potential means of reducing calorie intake, unlike other types of food labelling, PACE labelling may also promote participation in physical activity [15]. Unlike TLLs, PACE labelling does not require complex mental mathematical calculations to understand the full calorie content [16]. Evidence suggests that PACE labelling may influence food/drink choice [17], including among adolescents [18]. Most research in this area however has tested the effects of PACE food labels in laboratory settings/hypothetical food choice scenarios in adults and more real-world studies are needed to test the effectiveness of PACE food labels in reducing calorie intake and increase physical activity in both adults and children. Qualitative research has indicated that young people may prefer PACE labelling over other types of labelling [19].

As young people start to make independent decisions about what and where they eat, it is crucial that they are given understandable information to help them in their decision making. This study aimed to compare young people’s views (perceptions) and understanding of TLLs and PACE labels. Additionally, the study aimed to gather views about the possible implementation of PACE labelling such as preferences for locations and the types of food and drinks PACE labelling could be displayed on. Answers to these questions may help guide future health policy about the role of PACE labelling as a public health strategy.

## Methods

### Study procedure and participants

The study was approved by Loughborough University’s Ethics Approvals (Human Participants) Sub-Committee (reference: 1484). Ten secondary schools were recruited across the East and West Midlands, UK. The percentage of pupils eligible for free school meals was used as a proxy for school level socioeconomic position/deprivation [20]. Urban/rural description of schools was also collected [20].

Parental opt-out consent was used. Schools sent parents of eligible school children information about the study and details about how to opt out their child from participating. If parents did not opt-out within seven days their agreement was assumed. Cross-sectional data were collected between October 2020 to March 2021. Students in school years 8 to 13 (age 12 to 18) were invited to take part in the study. Teachers and administration staff sent students a link to an online questionnaire. When students opened the link, they were asked to read the study participant information sheet and thereafter if they were in agreement, to complete an assent/consent form (depending on their age).

### Measures

An online food label questionnaire was developed to explore adolescents’ views and understanding of food labels (in Additional file 1). The questionnaire was reviewed by a registered dietician on its design/content and dietetic accuracy. Nine adolescent members of the public also provided feedback on questionnaire length, ease of understanding and additional response options for food settings and food/drinks PACE labelling would be most useful on. The questionnaire was developed to be easily interpreted by young people. Pictorial examples of TLLs and PACE labels placed on/near food/drinks were included to help guide young people through the questionnaire. All questions that asked participants to rate their views/understanding used a 5-point Likert scale. All questions included a ‘don’t know’ response option and all demographic questions included a ‘prefer not to say’ option. Participants were asked to provide demographic data that included their age, school year group, gender and ethnicity.

Participants were asked about their understanding of the meaning of calories and whether they had previously seen TLLs on food and drinks. Those who answered ‘yes’, were asked where they had seen them from several food/drink setting options.

Participants rated their understanding of the information on TLLs and PACE labels from ‘very hard to understand’ to ‘very easy to understand’. Participants were also asked to rate from ‘none of it’ to ‘all of it’ how much of each label they understood and to select the label that was easier for them to understand (‘TLL’, ‘PACE label’ or ‘don’t know’) and an open-ended question explored why they chose the particular label.

Participants were asked to rate from ‘never’ to ‘always’ how often they look at TLLs and would look at PACE labels if they were placed on food/drinks. Participants who responded that they never look at the TLL and/or would never look at the PACE label were asked to choose up to three reasons why from a range of options (see Additional file 1 for further information). Participants were asked to rate how useful TLLs are and how useful PACE labels would be from ‘not at all useful’ to ‘extremely useful’.

Participants were asked to rate from ‘never’ to ‘always’ how often food labels prevent them (for the TLL) or would prevent them (for the PACE label) from buying unhealthy food and unhealthy drinks. Young people were asked to select the labelling system that would make it easier for them to choose healthy food and drinks on their own (‘TLL’, ‘PACE label’ or ‘don’t know’) and were asked an open-ended question why they chose that label. Two questions asked participants to select which type of label takes them less time to read and catches their attention the most (‘TLL’, ‘PACE label’ or ‘don’t know’) and then an open-ended question to explain their choices. One question asked participants whether PACE labelling on food and drinks would encourage them to do physical activity (‘yes’, ‘no’ or ‘don’t know’).

Participants who reported that PACE labels would be useful to some extent (from ‘slightly useful’ to ‘extremely useful’) were presented with two questions about their preferences for PACE labels. The first question asked participants to select up to four locations where it would be most useful to have PACE labels on food and drinks from a range of options. The second question asked participants to select up to four food/drinks PACE labels would be most useful on from a range of options.

At the end of the questionnaire, there was a call for volunteers to take part in qualitative interviews to discuss in more depth their views about food labels. Interviews explored views and understanding of, and preferences for, PACE labelling (not reported here).

### Data analyses

Quantitative data analyses were conducted using IBM SPSS version 25. Descriptive statistics were explored, and analyses conducted to assess associations between variables and to test differences in proportions. Pearson chi-square tests were conducted to: 1) explore relationships between TLLs and PACE labels on views and understanding; and 2) explore relationships between the views/understanding of food labels and demographic variables (age, gender and ethnicity). Prior to conducting these tests, responses of views and understanding of food labels, and demographics, were collapsed into two or three categories to allow for contingency tables to be produced. Binomial tests were conducted on responses to questions that asked participants to compare the food labels. Only participants who selected a label were included in the binomial tests. The null hypothesis was set at 50% for each label. Content analysis was used to summarise the free-text responses to the open-ended questions. Free-text responses were coded inductively into categories.

## Results

### Participant characteristics

The study questionnaire was sent to ~ 7,000 young people in school settings. A total of 808 responses were received (12% response rate), of which 54% were females. Table 1 summarises participant characteristics (gender, age and ethnicity). The age of participants ranged from 12 to 18 years (mean age 14.4 years, SD = 1.7). Schools were from affluent and deprived areas as represented by the percentage of pupils eligible for free school meals (schools ranged from 6 to 39%). Schools were from urban and rural areas.

**Table 1:** Participant characteristics

Characteristic	Total participants (*n* = 808)
**Gender, *n *(%)**	
Male (a boy)	340 (42.1)
Female (a girl)	436 (54)
Other	11 (1.4)
Prefer not to say/Missing	21 (2.6)
**Age (years), *n *(%)**	
12/13	272 (33.7)
14/15	292 (36.1)
16/17/18	218 (27)
Prefer not to say/Missing	26 (3.2)
**Ethnicity, *n *(%)**	
White	475 (58.8)
Asian	170 (21)
Black	58 (7.2)
Mixed	56 (6.9)
Other	17 (2.1)
Prefer not to say/Missing	32 (4)

### Understanding of calories

Seventy-one percent of participants (*n* = 575) correctly understood what calories are. Older participants and males were more likely to answer correctly (age: χ^2^ (2, *n* = 782) = 37.09, *p* < 0.001, *ϕ*_*c*_ = 0.22, gender: χ^2^ (1, *n* = 776) = 7.35, *p* < 0.01,* ϕ* = 0.1). Most participants (70%, *n* = 563) selected the correct number of calories in the example TLL presented, with older aged respondents, males and participants of white ethnicity more likely to answer correctly (age: χ^2^ (2, *n* = 782) = 26.71, *p* < 0.001, *ϕ*_*c*_ = 0.19, gender: χ^2^ (1, *n* = 776) = 6.92, *p* < 0.01, *ϕ* = 0.09, ethnicity: χ^2^ (1, *n* = 776) = 10.8, *p* = 0.001, *ϕ* = 0.12).

### Awareness of traffic light labels

Most young people (96%, *n* = 773) reported that they had previously seen TLLs on food and drinks. These participants had seen TLLs in supermarkets/shops (94%, *n* = 726) and in the home (72%, *n* = 558). To a lesser degree, participants also reported they had seen TLLs on packaged foods/drinks in coffee shops or cafés (55%, *n* = 427) and food/drinks bought from vending machines (52%, *n* = 402).

### Understanding of food labels

When asked to select the label that was easier for them to understand, a significantly higher proportion of participants selected the PACE label as easier to understand (*n* = 509), compared to the TLL (*n* = 233) (69% vs 31% respectively, *p* < 0.001). The free-text responses highlighted the main reasons why the PACE label was easier to understand is because they are considered simple (56%) and show physical activity information (38%). Participants who selected the TLL as easier to understand reported in their free-text responses this was because the TLL shows nutrient information (53%) and is simple (19%).

Sixteen percent of participants reported TLLs were very easy to understand, compared to 43% for PACE labels (Table 2). Of the total participants, 49% reported both labels easy to understand. There was a significant association between understanding of TLLs and PACE labels (χ^2^ (4, *n* = 808) = 41.53, *p* < 0.001, *ϕ*_*c*_ = 0.16), with 81% of those who found TLLs easy to understand, also finding PACE labels easy to understand. Conversely, 59% of those who found TLLs hard to understand, found PACE labels easy to understand. Only 2% of all participants reported both labels were hard to understand. Older participants found TLLs easier to understand than younger participants (χ^2^ (4, *n* = 782) = 13.22, *p* = 0.01, *ϕ*_*c*_ = 0.09).

**Table 2:** Understanding of food labels

**How hard or easy is it to understand the information on the labels?**		
	**TLL** ***n* = 808, *n *(%)**	**PACE label** ***n* = 808, *n *(%)**
Very hard to understand	11 (1.4)	19 (2.4)
Hard to understand	60 (7.4)	38 (4.7)
Neither hard nor easy to understand/Don’t know ^a^	247 (30.6)	151 (18.7)
Easy to understand	364 (45)	251 (31.1)
Very easy to understand	126 (15.6)	349 (43.2)
**How much of the label do you understand?**		
	**TLL** ***n* = 808, *n *(%)**	**PACE label** ***n* = 808, *n *(%)**
None of it/Don’t know ^b^	33 (4.1)	60 (7.4)
A little bit of it/Some of it	179 (22.2)	143 (17.7)
Most of it	370 (45.8)	170 (21)
All of it	226 (28)	435 (53.8)

^a^Don’t know responses: TLLs *n* = 36, PACE labels *n* = 33. Includes missing responses: TLLs *n* = 2, PACE labels *n* = 6

^b^Don’t know responses: TLLs *n* = 11, PACE labels *n* = 17. Includes missing responses: TLLs *n* = 1, PACE labels *n* = 3

### Frequency of use of food labels

Of participants who had seen TLLs on food and drinks before, 19% looked at TLLs often/always. Out of the total participants, 42% said they would look at PACE labels often/always if they were implemented (Table 3). There was a significant association between how often young people looked/would look at the labels (χ^2^ (4, *n* = 773) = 94.36, *p* < 0.001, *ϕ*_*c*_ = 0.25). Participants who looked at TLLs were more likely to say they would look at PACE labels. Specifically, 67% of participants who looked at TLLs often/always also reported that they would look at PACE labels often/always. Furthermore, 43% of participants who looked at TLLs rarely/sometimes said that they would look at PACE labels more often; and 68% who never looked at TLLs said they would look at PACE labels more often. Females and participants of non-white ethnicity were more likely to report that they would look at PACE labels often/always (gender: χ^2^ (2, *n* = 776) = 10.64, *p* < 0.01, *ϕ*_*c*_ = 0.12, ethnicity: χ^2^ (2, *n* = 776) = 9.82, *p* < 0.01, *ϕ*_*c*_ = 0.11). The most common reason why participants never/would never look at food labels is because they are not interested in making healthy choices.

**Table 3:** Frequency of use of food labels and main reasons why participants never/would never look at food labels

**How often do you/would you look at food labels to help you decide what food and drinks to buy or eat?**		
	**TLLs** ***n* = 773**^**a**^**, *n *(%)**	**PACE labels** ***n* = 808**^**b**^**, *n *(%)**
Never/Don’t know ^c^	199 (25.7)	132 (16.3)
Rarely/Sometimes	429 (55.5)	335 (41.5)
Often/Always	145 (18.8)	341 (42.2)
**What are the main reasons you never/would never look at the food labels?**		
	**TLLs** ***n* = 192**^**d**^**, *n *(%)**	**PACE labels** ***n* = 85**^**e**^**, *n *(%)**
I’m not interested in making healthy choices	71 (37)	29 (34.1)
I don’t have time/I would not have time	58 (30.2)	24 (28.2)
I never notice them/I would not notice them	56 (29.2)	23 (27.1)
I don’t buy food and drinks on my own	51 (26.6)	6 (7.1)
They look too complicated/They would look too complicated	32 (16.7)	20 (23.5)
I don’t understand them/I would not understand them	28 (14.6)	23 (27.1)
They are too small/They would be too small	10 (5.2)	4 (4.7)
I don’t think they are a good idea	Not an option	22 (25.9)

^a^Number of participants who have seen TLLs on food and drinks before

^b^Total number of participants in study

^c^Don’t know responses: TLLs *n* = 7, PACE labels *n* = 47

^d^Number of participants who said they would never look at TLLs

^e^Number of participants who said they would never look at PACE labels

Main reasons never look at TLLs: other responses *n* = 11, don’t know responses *n* = 24

Main reasons would never look at PACE labels: other responses *n* = 14, don’t know responses *n* = 13

### Perceived usefulness of food labels

Nineteen percent of participants reported TLLs are very useful/extremely useful and 29% reported PACE labels would be very useful/extremely useful (Table 4). Most young people (83%) said that PACE labels would be useful to some extent. There was a significant association between perceived usefulness of TLLs and PACE labels (χ^2^ (4, *n* = 808) = 172.88, *p* < 0.001, *ϕ*_*c*_ = 0.33). About half of participants (51%) who reported TLLs as very useful/extremely useful, also reported the same for PACE labels. Furthermore, 49% of the participants who reported TLLs as not at all useful reported PACE labels as more useful. Females were more likely to report PACE labels as very useful/extremely useful (χ^2^ (2, *n* = 776) = 7.8, *p* < 0.05, *ϕ*_*c*_ = 0.1).

**Table 4:** Perceived usefulness of food labels and perceived effect of food labels on food/drink choice

**How useful are TLLs/would PACE labels be to help you decide what food and drinks to buy or eat?**		
	**TLLs** ***n* = 808, *n *(%)**	**PACE labels** ***n* = 808, *n *(%)**
Not at all useful/Don’t know ^a^	144 (17.8)	141 (17.5)
Slightly useful/Somewhat useful	507 (62.7)	433 (53.6)
Very useful/Extremely useful	157 (19.4)	234 (29)
**Do/would food labels stop you buying unhealthy food?**		
	**TLLs** ***n* = 581**^**b**^**, *n *(%)**	**PACE labels** ***n* = 723**^**c**^**, *n *(%)**
Never/Don’t know ^d^	106 (18.2)	146 (20.2)
Rarely/Sometimes	406 (69.9)	419 (58)
Often/Always	69 (11.9)	158 (21.9)
**Do/would food labels stop you buying unhealthy drinks?**		
	**TLLs** ***n* = 581**^**b**^**, *n *(%)**	**PACE labels** ***n* = 723**^**c**^**, *n *(%)**
Never/Don’t know ^e^	132 (22.7)	156 (21.6)
Rarely/Sometimes	363 (62.5)	391 (54.1)
Often/Always	86 (14.8)	176 (24.3)

^a^Don’t know responses: TLLs *n* = 36, PACE labels *n* = 53. Includes missing responses: TLLs *n* = 3, PACE labels *n* = 0

^b^Number of participants who look at TLLs to some extent (includes don’t know responses)

^c^Number of participants who would look at PACE labels to some extent (includes don’t know responses)

^d^Don’t know responses: TLLs *n* = 18, PACE labels *n* = 64. Includes missing responses: TLLs *n* = 0, PACE labels *n* = 3

^e^Don’t know responses: TLLs *n* = 19, PACE labels *n* = 66. Includes missing responses: TLLs *n* = 1, PACE labels *n* = 1

### Perceived effect of food labels on food/drink choice

A higher proportion (52%, *n* = 380) of participants selected the PACE label as the label that would make it easier for them to choose healthy food and drinks on their own, compared to the TLL (48%, *n* = 357). These proportions were not significantly different, *p* = 0.418. The free-text responses highlighted that the PACE label would make it easier to choose healthy food and drinks because they are simple/easier to understand (43%) and they show physical activity information (41%). The main reasons why young people chose the TLL is because they show nutrient information (54%) and have colours (13%).

Of participants who said they looked at TLLs, some said these labels stop them buying unhealthy food and drinks (12% and 15% of participants, respectively) often/always (Table 4). Of participants who said they would look at PACE labels, 22% believed they would stop them buying unhealthy food often/always and 24% said they would stop them buying unhealthy drinks often/always (Table 4).

There was a significant association between the frequency at which TLLs stopped young people buying unhealthy food/drinks and how often PACE labels would (unhealthy food: χ^2^ (4, *n* = 552) = 92.46, *p* < 0.001, *ϕ*_*c*_ = 0.29, unhealthy drinks: χ^2^ (4, *n* = 552) = 174.9, *p* < 0.001, *ϕ*_*c*_ = 0.4). The participants who reported TLLs stopped them buying unhealthy food/drinks were likely to say the same for PACE labels. Of participants who said that TLLs never stopped them buying unhealthy food, 67% reported PACE labels would stop them buying unhealthy food more often. Furthermore, of participants who said that TLLs never stopped them buying unhealthy drinks, 58% reported PACE labels would stop them buying unhealthy drinks more often.

### Views on aesthetics of labels

A significantly higher proportion of participants selected the PACE label (*n* = 623) as the label that would take less time for them to read, compared to the TLL (*n* = 132) (83% vs 17% respectively, *p* < 0.001). Reasons why the PACE label would take less time to read is because it shows less information (45%) and is simple (32%). Reasons given why the TLL takes less time to read is that it has colours (27%) and is easier to understand (22%).

Compared to the TLL (*n* = 255), a significantly higher proportion of participants selected the PACE label (*n* = 463) as the type of label that catches their attention the most (36% vs 64% respectively, *p* < 0.001). The PACE label would catch attention the most because it is simple/easier to read (36%) and is bigger (29%). The main reasons why the TLL would catch attention the most is because of the colours (40%) and that it shows more information (23%).

### PACE labels and physical activity

Fifty percent of participants reported that PACE labels would encourage them to participate in physical activity, 24% reported PACE labels would not encourage them to participate, and 25% reported ‘don’t know’.

### Preferences for PACE labels

Participants selected a range of eating locations where PACE labels would be most useful, and a range of food/drinks PACE labels would be most useful on. See Table 5.

**Table 5:** Preferences for PACE labels

**Where do you think it would be most useful to have PACE labels on food and drinks?**	
	**Total *n* = 667**^**a**^ ***n *(%)**
Supermarkets/shops	599 (89.8)
Fast food places	506 (75.9)
Vending machines	375 (56.2)
School canteen	361 (54.1)
Coffee shops/cafés	193 (28.9)
Restaurants	179 (26.8)
**If PACE labels were put on food and drinks, what food and drinks would it be most useful to see them on?**	
	**Total *n* = 667**^**a**^ ***n *(%)**
**Snacks**	
Chocolate, sweets	449 (67.3)
Sweet biscuits/cookies	270 (40.5)
Sweet cakes, pastries, pies	255 (38.2)
Crisps	164 (24.6)
**Drinks**	
Sugary fizzy drinks	421 (63.1)
Energy drinks	220 (33)
Milkshakes	68 (10.2)
**Meal items**	
Burgers, chicken nuggets, kebabs	348 (52.2)
Pizza	145 (21.7)
Chips, fries	114 (17.1)
Pasta	37 (5.5)
Sandwiches	29 (4.3)

^a^Number of participants who said PACE labels would be useful to some extent (*n* = 667)

Places where it would be most useful to have PACE labels on food and drinks: other responses *n* = 10, don’t know responses *n* = 21

Food and drinks it would be most useful to see PACE labels on: other responses *n* = 15, don’t know responses *n* = 13

## Discussion

The present study aimed to examine the views of young people about, and understanding of, food labels. Findings in this study indicate that PACE labels may be easier to understand and more useful than TLLs. PACE labels may help some young people choose healthy food and drinks on their own. It was perceived that PACE labels could be useful in a range of food settings and on a range of food/drinks.

### Understanding of food labels

Though the aim of food labelling is to inform the public about what they are eating and drinking, consistent with other research [13], our findings indicate that adolescents may find TLLs difficult to understand. Some young people also appeared to not understand the concept of calories, and therefore may find it difficult to comprehend this information when displayed on food labels. Findings indicate that some young people may find PACE labels easier to understand than TLLs. The main reasons given by participants for this is because PACE labels are simple and show physical activity information. PACE labelling contextualises the energy content of food/drinks which may help young people understand the context of eating/drinking unhealthily such as how it relates to energy balance. PACE labelling relies less on hypothetical thinking, which some children and adolescents may not yet have developed [14], making PACE labelling information more accessible to young people.

### Usefulness and impact of food labels on food/drink choice

Consistent with previous research suggesting that nutrition labels may not be effective in altering food purchasing or consumption [11, 12], this study found that the use of TLLs among adolescents was low. Participants reported that they would look at PACE labels more often than TLLs and overall, perceived PACE labels as more useful. This suggests a preference for PACE labels in young people, supporting findings by Evans et al. (2016) who found that young people may prefer PACE labelling over other types of labelling [19]. Our findings indicate that PACE labels may make it easier for some young people to choose healthy food and drinks on their own. PACE labels may prevent young people buying unhealthy food and drinks e.g. discretionary foods, which could contribute to reducing overconsumption behaviour and thus leading to a reduction in overweight and obesity, if such behaviour changes are sustained.

It is interesting that views were divided about the type of labelling that would make it easier for participants to choose healthier food and drinks. This result may add to the case that placing both the TLL and PACE label on packaged food and drinks, or a ‘hybrid’ incorporating the most important elements of both types of labelling, may be of benefit. This could serve the needs of most young people by displaying nutrient information to those who need/want it, as well as providing contextual information on energy content in food/drinks. On unpackaged food and drinks, PACE labelling could complement absolute calorie labelling to provide young people with more information about energy.

The main reason why some participants reported that they do not look at food labels is because they are not interested in making healthy choices. This is of real concern because this view could lead to the overconsumption of calories. Moreover, it is critically important that young people are aware of the importance to their health of the impact of eating a healthy diet. It may be that these young people would not look at any type of food label and further research on this question would be worthwhile. It may be that another type of intervention is required to help young people choose healthier food and drinks, particularly in contexts such as school canteen environments, where young people are making food selections every day. This also highlights that food labels are one part of a larger strategy to promote healthy eating and prevent/reduce the number of children living with obesity. Nevertheless, the present findings suggest focusing efforts on making food labels simple, quick to read and noticeable would be of benefit for many young people. Further research that assesses the merits of PACE labelling in young people, particularly in contexts such as school canteens where young people are making their own decisions about food, is required. Of note here, some concerns have been raised that PACE labelling may have an adverse effect by promoting eating disorders [21]. Though there is no evidence that this is the case, this is an important question that future research needs to address.

### PACE labels and physical activity

Research indicates that many young people are not doing enough physical activity each day [3]. This increases the risk of childhood obesity, as well as several other diseases in later life. As well as helping people to make healthier food choices, it has been suggested that PACE labels may also offer the opportunity to continually remind or nudge people to participate in regular physical activity [15]. This means that PACE labelling could have additional benefits over other types of labelling. Findings in this study indicate that PACE labelling could encourage some young people to do more physical activity, reducing their risk of obesity and other diseases. It has been found that PACE labelling may increase physical activity behaviour [15]. Future studies are required to test the effects of PACE labelling on physical activity behaviour in young people.

### Preferences for PACE labels

Results indicated that adolescents perceive PACE labels could be useful in settings that sell packaged food and drinks (e.g. shops) and those that sell unpackaged food and drinks (e.g. fast food outlets and coffee shops), highlighting the versatility of PACE labelling. Furthermore, adolescents perceived PACE labels could be useful when displayed on discretionary products such as confectionary and sugary fizzy drinks. This is an encouraging result given that discretionary products can lead to the excess consumption of calories [22]. There is evidence to suggest that PACE labelling may influence food/drink choice [17, 18] and further research now needs to be undertaken to assess if this is the case in real-world settings.

### Strengths and limitations of the study

This is the first study to explore the views and understanding of food labels, comparing the TLL and PACE label, in young people. There was a large ethnically diverse sample of young people and schools were recruited from a range of locations in the East and West Midlands of England. The questionnaire benefited from expert and public involvement in its development. Free-text responses from young people about food labels supplemented quantitative findings providing broader contextual information to participants’ responses.

The results of this study should also be interpreted in light of some limitations. The study was conducted online during the COVID-19 pandemic when social distancing requirements resulted in closures of schools to most students. This likely impacted the number of responses received. Adolescents with a higher interest in nutrition/food labelling may have been more likely to participate in the study, compared with their less interested counterparts, although we had strategies to reduce the likelihood of this occurring (e.g. public involvement in questionnaire development and asking schools to send reminders to students about the questionnaire). There is also the possibility that participants offered socially desirable responses. To gather views about PACE labels, questions were framed hypothetically as these labels are not implemented in any country and this meant that awareness of PACE labels in food settings could not be examined. Additionally, the observational nature of the study means that causal explanations cannot be made. Deprivation status at the participant level was not explored in this study. Despite the limitations of the research, the findings provide important information about the views of food labels among adolescents in the UK. These findings could be used to inform future research in food labelling interventions.

## Conclusion

PACE labels may be easier for adolescents to understand than TLLs, and may be more appealing and impactful on decisions about food and drink consumption. PACE labelling may be a promising strategy to help adolescents choose healthier food and drinks therefore reducing the excess amounts of energy consumed in this population. Further research testing PACE labelling and understanding its impact in the adolescent population in real-world settings is warranted.

## Supplementary Information

**Additional  file 1.** 

## Data Availability

Data will be deposited in an appropriate data repository once the programme of research has been completed.

## Abbreviations

IBM SPSS: IBM Statistical Package for Social Sciences
PACE: Physical activity calorie equivalent
TLL: Traffic light label
UK: United Kingdom
