# Supplementary material for: Examining young people’s views and understanding of traffic light and physical activity calorie equivalent (PACE) food labels
Source: BMC Public Health. 2023 Jun 14;23:1143. doi: 10.1186/s12889-023-16019-6 (PMC10264867; doi:10.1186/s12889-023-16019-6)
Supplement: Supplementary file 1 — Additional file 1. [file 12889_2023_16019_MOESM1_ESM.pdf]

Additional file 1: Nutrition label questionnaire

The views of young people about nutrition labels on food and drinks

How to fill in this questionnaire

- Please answer the questions by putting a tick in the box ☐ by the answer you want to give, like this:

Yes	No
☑	☐

- Below some of the boxes are arrows and instructions. They tell you which question to answer next. If there is nothing there, just go to the next question.

Yes	No
☐	☐
↓	↓
go to **Q2**	go to **Q3**

- Sometimes you might be asked to fill in more about your answer in a box, like the one below:

**Write in…**

Section 1: This section is about the way some food is currently labelled in shops.

Below is a picture of a label on packaging for a pizza. It is an example of a label used on some food and drinks. It is called a traffic light label.

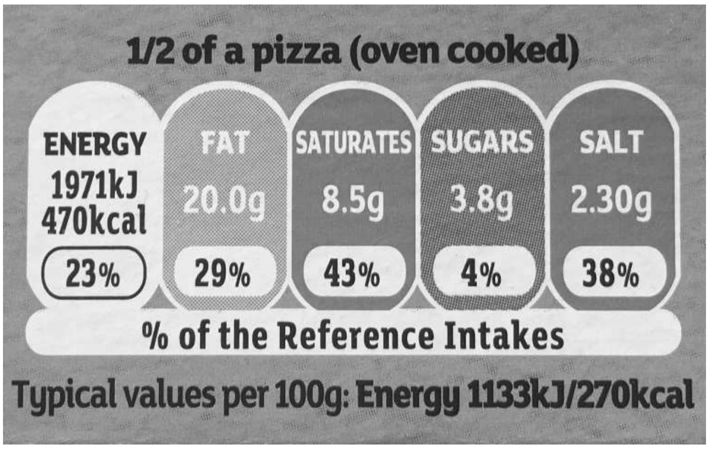

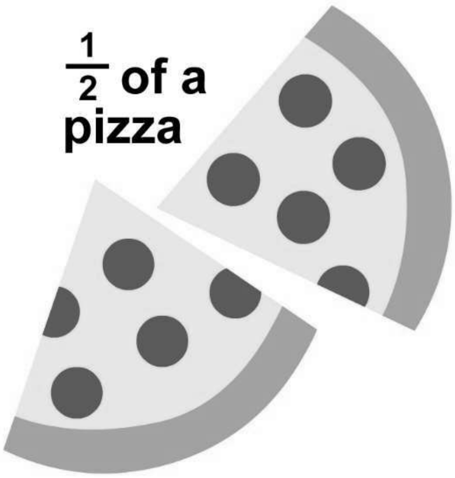

You might have seen this type of traffic light label on food and drinks in shops, at school or at home. We would like to ask you some questions about this type of label. If you have not seen this type of label before, don't worry. Just answer the questions as best you can. Thank you.

Q1. Have a look at the food traffic light label above. How much of the traffic light label do you understand?

None of it	A little bit of it	Some of it	Most of it	All of it	Don't know
☐	☐	☐	☐	☐	☐

Q2. How hard or easy is it to understand the information on food traffic light labels (like the one above)?

Very hard to understand	Hard to understand	Neither hard nor easy to understand	Easy to understand	Very easy to understand	Don't know
☐	☐	☐	☐	☐	☐

Q3. Which colour on a food traffic light label shows the food/drink is high in fat, saturates, sugar or salt?

Green	Amber	Red	Don't know
☐	☐	☐	☐

Q4. What are **calories**?

The weight in our bodies	Minerals that make our bones strong	Energy we get from food and drinks	Fat found in food and drinks	Don't know
☐	☐	☐	☐	☐

Q5. Looking at the traffic light label below, how many calories does half a pizza contain?

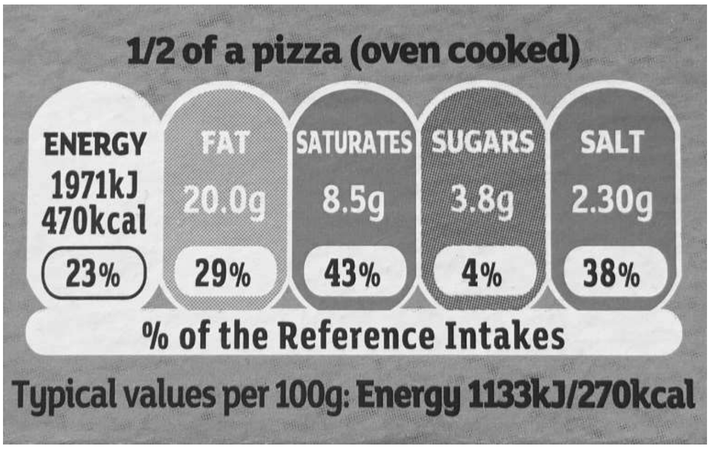

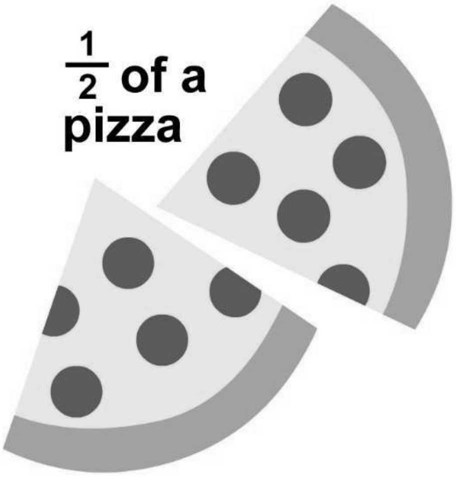

1971	470	1133	270	Don't know
☐	☐	☐	☐	☐

Q6. Have you seen traffic light labels on food and drinks before?

Yes I have	No I haven't	Don't know
☐	☐	☐
↓	↓	↓
go to **Q7**	go to **Q12**	go to **Q12**

Q7. Where have you seen food traffic light labels?

Please tick all that apply

- At home ☐
- Supermarkets/ shops ☐
- Packaged foods/drinks in coffee shops or cafés ☐
- Food/drinks I have bought from vending machines ☐
- Other ☐ → **Write in…**
- Don't know ☐

Q8. How often do you look at food traffic light labels to help you decide what food and drinks to buy or eat?

Never	Rarely	Sometimes	Often	Always	Don't know
☐	☐	☐	☐	☐	☐
go to **Q9**	go to **Q10**	go to **Q10**	go to **Q10**	go to **Q10**	go to **Q10**

Q9. What are the main reasons you never look at food traffic light labels?

Please tick **no more** than three

- I don't understand them ☐
- I don't have time ☐
- I don't buy food and drinks on my own ☐
- I never notice them ☐
- They are too small ☐
- They look too complicated ☐
- I'm not interested in making healthy choices ☐
- Other ☐ → **Write in…**
- Don't know ☐

Please go to **Q12**

Below is another example of a traffic light label, on a fizzy soft drink**.**

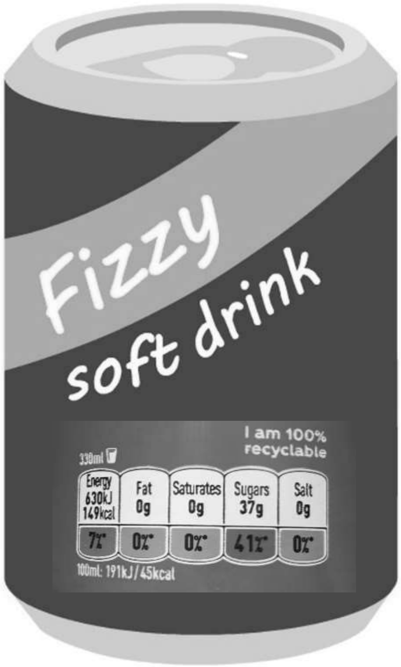

Q10. Do food traffic light labels stop you buying *unhealthy* **food?**

Never	Rarely	Sometimes	Often	Always	Don't know
☐	☐	☐	☐	☐	☐

Q11. Do food traffic light labels stop you buying *unhealthy* **drinks**? (e.g. cola, milkshakes)

Never	Rarely	Sometimes	Often	Always	Don't know
☐	☐	☐	☐	☐	☐

Q12. How useful are traffic light labels to help you decide what food and drinks to buy or eat?

Not at all useful	Slightly useful	Somewhat useful	Very useful	Extremely useful	Don't know
☐	☐	☐	☐	☐	☐

Section 2: This section asks you about a new way to label food and drinks called **p**hysical **a**ctivity **c**alorie **e**quivalent labelling. Its short name is **PACE** labelling.

PACE labelling shows people how many minutes of physical activity are needed to burn off calories in food and drinks. For example, if there are 470 calories in half a pizza, the box would show that to burn off the calories it would take about 47 minutes of running. Have a look at the pictures below to show you some examples of PACE labelling.

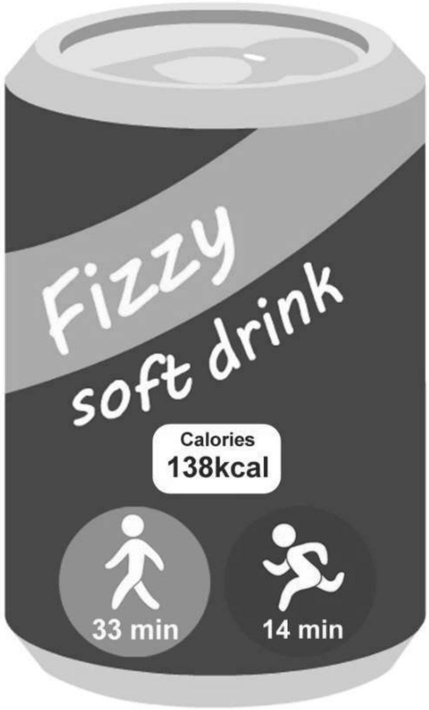

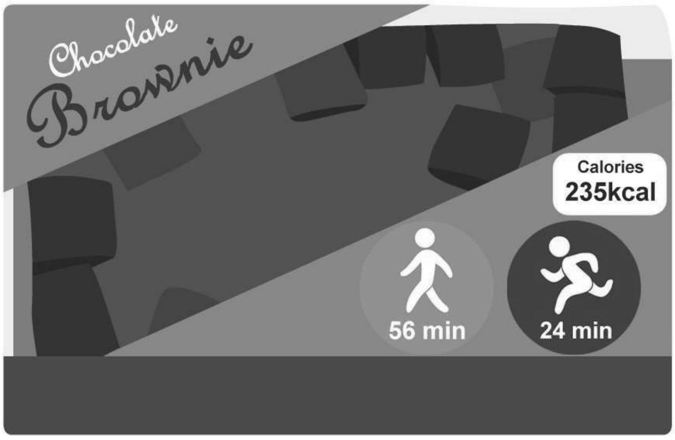

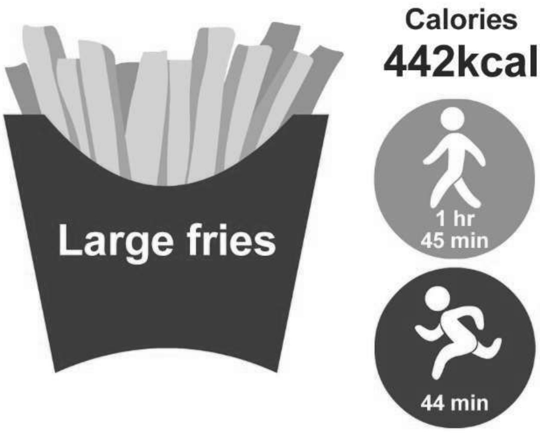

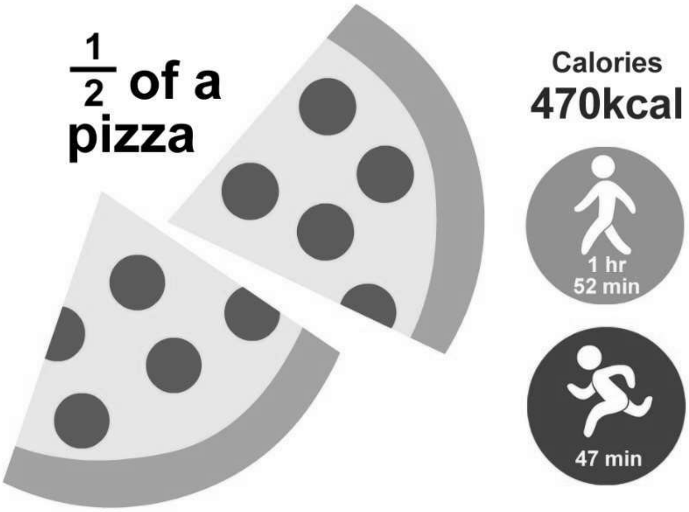

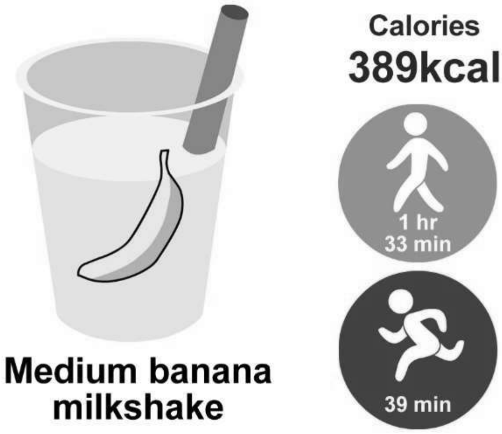

Some people have said this type of labelling:

- is easier to understand than traffic light labels.
- can help people choose healthy foods/drinks and avoid unhealthy foods/drinks.
- can encourage people to be more physically active.

We are interested in what <u>you</u> think about this.

Q1. Looking at the examples of PACE labels, how much of the label do you understand?

None of it	A little bit of it	Some of it	Most of it	All of it	Don't know
☐	☐	☐	☐	☐	☐

Q2. How hard or easy is it to understand the information on the PACE labels?

Very hard to understand	Hard to understand	Neither hard nor easy to understand	Easy to understand	Very easy to understand	Don't know
☐	☐	☐	☐	☐	☐

Q3. If PACE labels were put on food/drinks, how often would you look at them to help you decide what food and drinks to buy or eat?

Never	Rarely	Sometimes	Often	Always	Don't know
☐	☐	☐	☐	☐	☐
↓	↓	↓	↓	↓	↓
go to **Q4**	go to **Q5**	go to **Q5**	go to **Q5**	go to **Q5**	go to **Q5**

Q4. What are the main reasons you would never look at PACE labels?

Please tick **no more** than three

I would not understand them	☐	I would not have time	☐
I don't buy food and drinks on my own	☐	I would not notice them	☐
They would be too small	☐	They would look too complicated	☐
I'm not interested in making healthy choices	☐	I don't think they are a good idea	☐
Other	☐	Don't know	☐

↓

Write in…

Please go to **Q7**

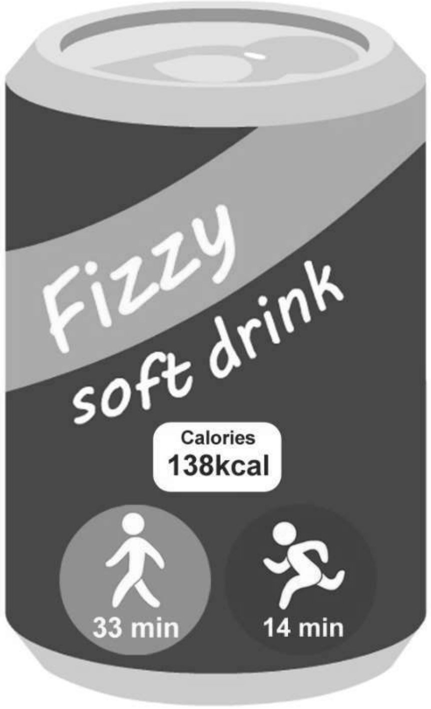

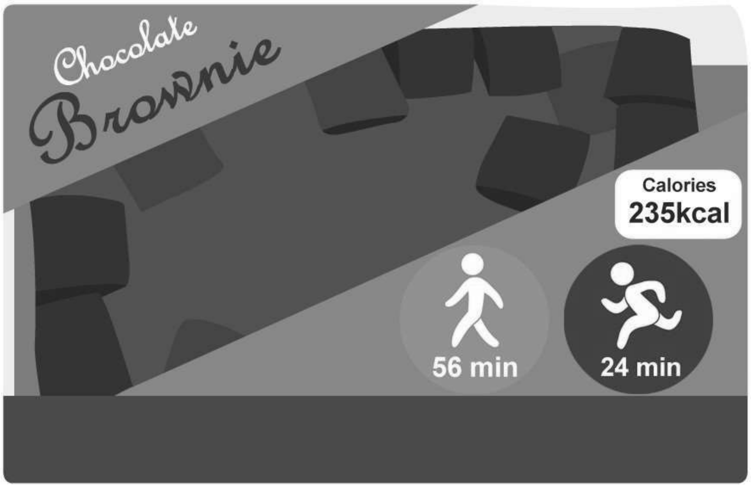

Q5. Would PACE labels stop you buying *unhealthy* **food**?

Never	Rarely	Sometimes	Often	Always	Don't know
☐	☐	☐	☐	☐	☐

Q6. Would PACE labels stop you buying *unhealthy* **drinks**? (e.g. cola, milkshakes)

Never	Rarely	Sometimes	Often	Always	Don't know
☐	☐	☐	☐	☐	☐

Q7. If you saw PACE labels on food and drinks would it encourage you to do physical activity? (e.g. walking, running, dancing, swimming)

Yes	No	Don't know
☐	☐	☐

Q8. How useful would PACE labels be to help you decide what food and drinks to buy or eat?

Not at all useful	Slightly useful	Somewhat useful	Very useful	Extremely useful	Don't know
☐	☐	☐	☐	☐	☐
↓	↓	↓	↓	↓	↓
go to **Section 3**	go to **Q9**	go to **Q9**	go to **Q9**	go to **Q9**	go to **Section 3**

Q9. Where do you think it would be **most** useful to have PACE labels on food and drinks?

Please tick **no more** than four.

Supermarkets/shops	☐	School canteen	☐
Restaurants	☐	Fast food places	☐
Coffee shops/cafés	☐	Vending machines	☐
Other	☐ ↓	Don't know	☐

Write in…

Q10. If PACE labels were put on food and drinks, what food and drinks would it be **most** useful to see them on?

Please tick **no more** than four.

Sweet biscuits/cookies	☐	Sweet cakes, pastries, pies	☐
Chocolate, sweets	☐	Crisps	☐
Burgers, chicken nuggets, kebabs	☐	Chips, fries	☐
Pizza	☐	Pasta	☐
Sandwiches	☐	Sugary fizzy drinks	☐
Energy drinks	☐	Milkshakes	☐
Other	☐ ↓	Don't know	☐

Write in…

Section 3: This section asks you about both the traffic light labels and PACE labels we have talked about.

Q1. Which label is easier for you to understand?

Please tick **one**

Traffic light label ☐ PACE label ☐ Don't know ☐

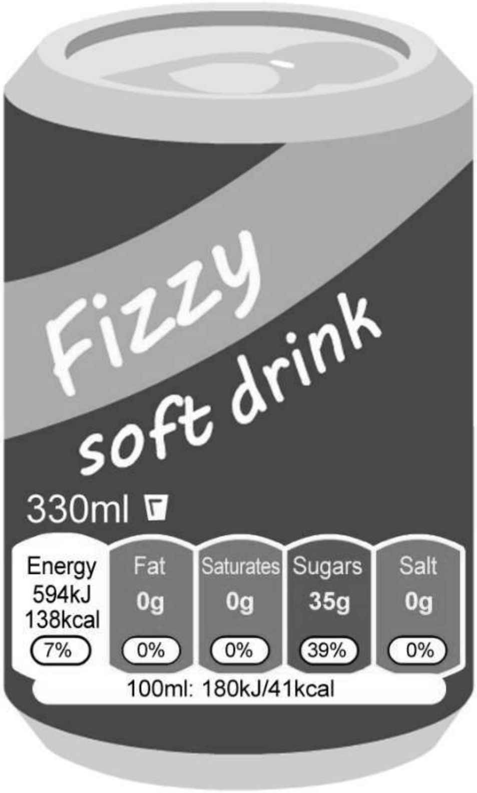

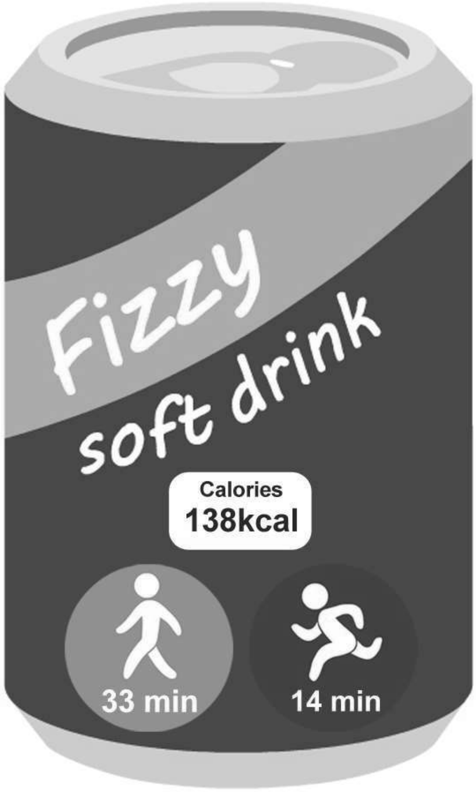

Why?

**Write in…**

Q2. Which label would make it easier for you to choose healthy food and drinks on your own?

Please tick **one**

Traffic light label ☐

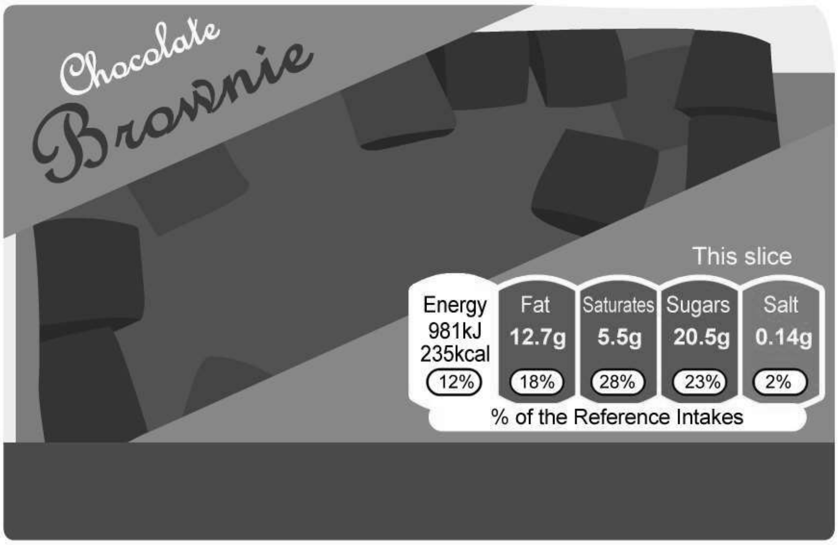

PACE label ☐

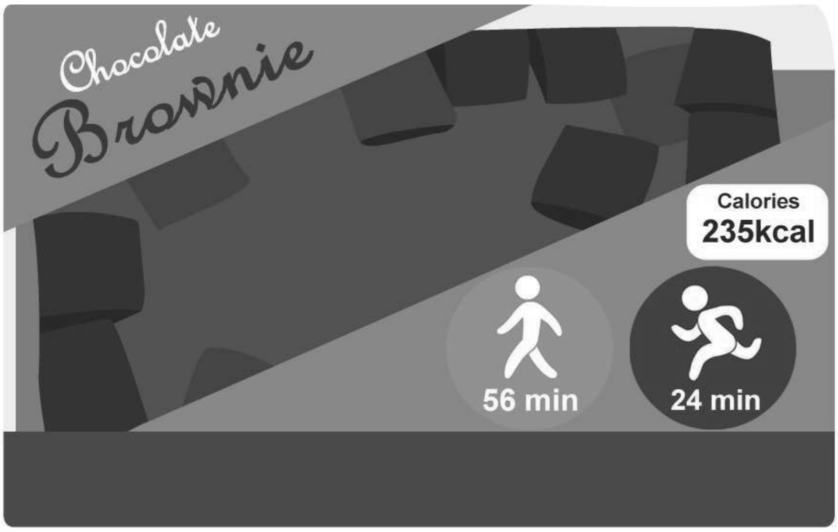

Don't know ☐

Why?

**Write in…**

Q3. Which label would take less time for you to read?

Please tick **one**

Traffic light label ☐ PACE label ☐ Don't know ☐

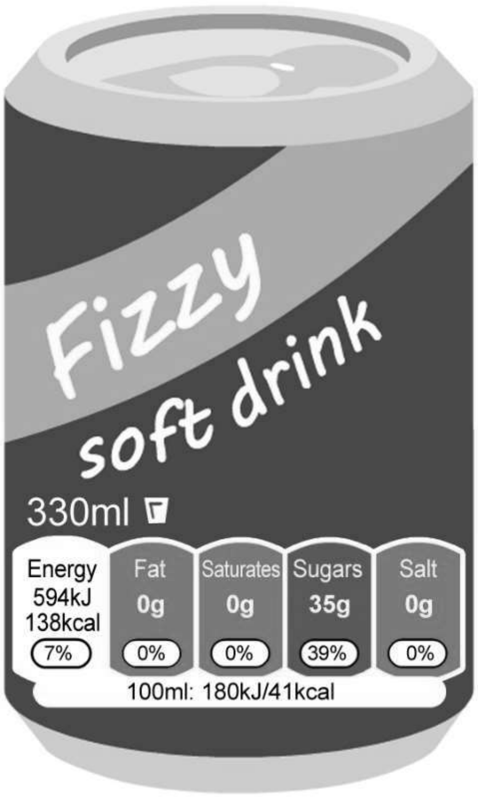

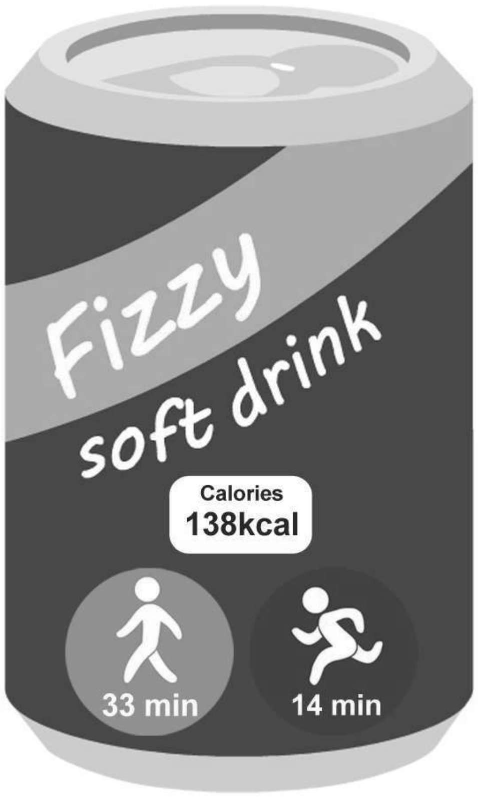

Why?

**Write in…**

Q4. Which label catches your attention the most?

Please tick **one**

Traffic light label ☐

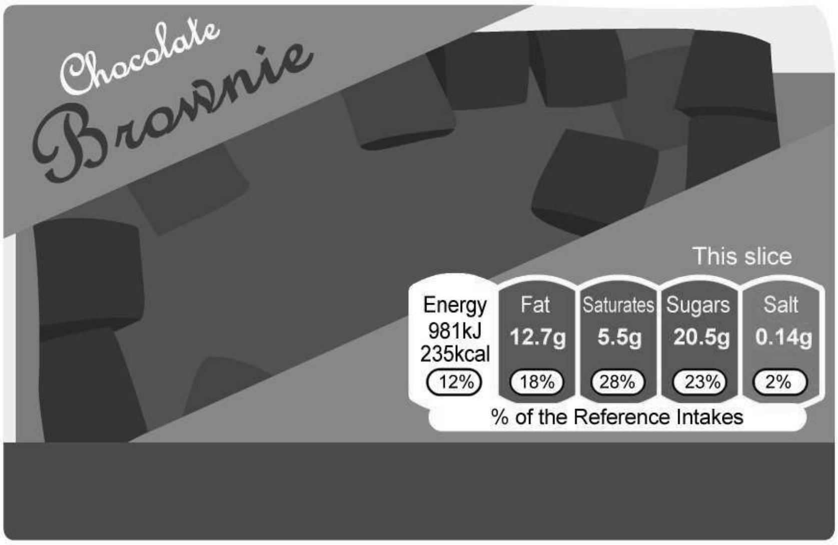

PACE label ☐

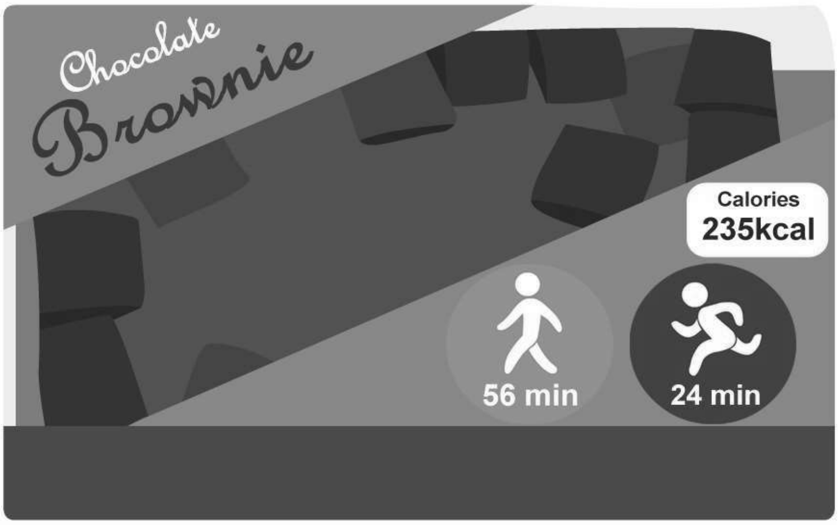

Don't know ☐

Why?

**Write in…**

Q5. Are there any other comments you would like to make about anything you have seen in this questionnaire?

Write in…

Section 4: About you

What is your school year group?

Year 8	Year 9	Year 10	Year 11	Year 12	Year 13	Prefer not to say
☐	☐	☐	☐	☐	☐	☐

What is your age?

12	13	14	15	16	17	18	Prefer not to say
☐	☐	☐	☐	☐	☐	☐	☐

What is your gender?

Male (a boy)	Female (a girl)	Other	Prefer not to say
☐	☐	☐	☐

Which of these best describes your ethnicity?

White	Asian	Black	Mixed	Other	Prefer not to say
☐	☐	☐	☐	☐	☐

IF COMPLETING FACE-TO-FACE IN CLASSES:

Are you interested in talking to Natalia about your views of food labels and how they could be improved? If you are interested, please let Natalia know at the end of the session.

Thank you for filling in this questionnaire!

IF COMPLETING ONLINE:

Participants needed

Are you interested in talking to Natalia (the researcher from Loughborough University) about your views of food labels and how they could be improved?

If you **are** interested, please fill in your details below and then press 'Next' to submit the questionnaire. If selected, your parent/guardian will be contacted.

If you **are not** interested, please just press 'Next' to submit the questionnaire.

What is your full name?____

What is your school year group?

Year 8	Year 9	Year 10	Year 11	Year 12	Year 13
☐	☐	☐	☐	☐	☐

What is your school tutor group?______________________________

Please press 'Finish' to submit the questionnaire

Thank you for filling in this questionnaire!
